# Letter to the Editor Regarding “Lateral Approach and Combined Lateral and Anteromedial Approach for Surgical Treatment of Terrible Triad of Elbow: A Meta-Analysis.”

**DOI:** 10.30476/BEAT.2020.86013

**Published:** 2020-07

**Authors:** Uttam Chand Saini, Deepak Neradi, Deepak Kumar, Vikas Bacchal, Praveen Sodavarapu, Akshay Shetty

**Affiliations:** 1 *Department of Orthopaedics, Post Graduate Institute of Medical Education and Research, Sector 12, Chandigarh 160012, India*

**Dear editor,**

We read with great interest the article** “**Lateral approach versus combined lateral and anteromedial approach for surgical treatment of terrible triad of the elbow: A meta-analysis” by Meena *et al*., [1]. They carried out this meta-analysis to find out the functional outcomes and the risk of complications in the terrible triad of elbow (TTIE) treated by two different approaches. They concluded that combined lateral and medial approach (CML) had significantly more elbow range of motion (ROM), forearm rotation, higher mayo elbow performance score (MEPS) but at the cost of significantly increased the operative time. 

Our first observation is that authors included four studies for this meta-analysis, of which study by Li *et al*., [2] is series of arthroscopic assisted surgeries and rest three were open surgical procedures that make the comparison heterogeneous. We also noted the discrepancy in the author’s conclusions and forest plots diagrammatic representation (showing favourable outcome with lateral approach). Moreover, the authors failed to include the study by Pierrart *et al*., [3] and Liu *et al*., [4], both compared the two approached for terrible triad injuries of elbow. When we analysed the data after adding these studies and removing arthroscopic study, we found that the total patients in the lateral only approach were 185 and 178 in the CML group. No significant difference was found in the functional outcome between the two approaches. The lateral only approach was associated with a shorter time duration (*p*=0.03) and fewer number of complications (*p*=0.02). So, the conclusion of the meta-analysis is questionable. The new conclusion that can be drawn is that majority of TTIE can be operated by the lateral only approach due to shorter time duration and fewer complications with no difference in the functional outcomes between the two approaches.

## Conflict of Interest:

None declared.
